# Stanford E. Chaille Jubilee

**Published:** 1908-04

**Authors:** 


Stanford E. Chaille Jubilee.—The Alumni of the Medical Department of Tulane University, New Orleans, purpose giving a Stanford E. Chaille Jubilee to celebrate the anniversary of the fiftieth year of teaching service of the honored and respected Professor and Dean of the Medical Department, to take place May 19 and 20, 1908. Every alumnus of the historic old college is invited and urged to participate in the exercises. The details will be announced later, and full program will be published in the May number of the Texas Medical Journal. Meantime, Professor Isadore Dyer, of the Faculty, and Chairman of Committee (P. O. Drawer 261, New Orleans), will be pleased to have the alumni write him indicating their intention to be present. The Texas Committee of Alumni selected to receive contributions to the Chaille Memorial Fund are Drs. Geo. H. Lee, Galveston; Amos Graves, Sr., San Antonio; O. L. Norsworthy, Houston; J. J. Dean, Waco, and W. W. Samuels, Dallas, either of whom will forward blanks on request to be filled and sent with contributions. Prof. Chaille's name will go down to posterity in company with that of Virchow, Flint, Pasteur and other great teachers.

